# Clinical Development of a Cytomegalovirus DNA Vaccine: From Product Concept to Pivotal Phase 3 Trial

**DOI:** 10.3390/vaccines1040398

**Published:** 2013-09-25

**Authors:** Larry R. Smith, Mary K. Wloch, Jennifer A. Chaplin, Michele Gerber, Alain P. Rolland

**Affiliations:** 1 Vical Incorporated, 10390 Pacific Center Court, San Diego, California, CA 92121, USA; E-Mails: mwloch@vical.com (M.K.W.); jchaplin@vical.com (J.A.C.); arolland@vical.com (A.P.R.); 2 Astellas Pharma Global Development, Inc., 1 Astellas Way, Northbrook, IL 60062, USA; E-Mail: michele.gerber@astellas.com

**Keywords:** plasmid DNA vaccine, cytomegalovirus (CMV), glycoprotein B (gB), phosphoprotein 65 (pp65), poloxamer CRL1005, benzalkonium chloride (BAK), hematopoietic cell transplant (HCT), CMV end organ disease (EOD)

## Abstract

2013 marks a milestone year for plasmid DNA vaccine development as a first-in-class cytomegalovirus (CMV) DNA vaccine enters pivotal phase 3 testing. This vaccine consists of two plasmids expressing CMV antigens glycoprotein B (gB) and phosphoprotein 65 (pp65) formulated with a CRL1005 poloxamer and benzalkonium chloride (BAK) delivery system designed to enhance plasmid expression. The vaccine’s planned initial indication under investigation is for prevention of CMV reactivation in CMV-seropositive (CMV^+^) recipients of an allogeneic hematopoietic stem cell transplant (HCT). A randomized, double-blind placebo-controlled phase 2 proof-of-concept study provided initial evidence of the safety of this product in CMV^+^ HCT recipients who underwent immune ablation conditioning regimens. This study revealed a significant reduction in viral load endpoints and increased frequencies of pp65-specific interferon-γ-producing T cells in vaccine recipients compared to placebo recipients. The results of this endpoint-defining trial provided the basis for defining the primary and secondary endpoints of a global phase 3 trial in HCT recipients. A case study is presented here describing the development history of this vaccine from product concept to initiation of the phase 3 trial.

## 1. Introduction

### 1.1. DNA Vaccine Historical Context

The seminal studies of the DNA vaccine field, published in the early 1990s, demonstrated that administration of plasmid DNA to mice resulted in protein expression of encoded transgenes [1], induction of antibody responses [2], and protection from pathogenic challenge [3]. Following these and other nonclinical proof of concept studies, the first-in-human phase 1 clinical trials were conducted during the mid-to-late 1990s in HIV-1-infected and normal healthy volunteers with DNA vaccines encoding HIV-1 and malaria antigens, respectively [4,5,6]. Since then, DNA vaccines against other infectious disease agents have completed phase 1 and in some cases phase 2 testing including: anthrax, CMV, dengue, Ebola, hepatitis B virus, hepatitis C virus, herpes simplex virus-2, human papillomaviruses, seasonal and pandemic influenza viruses, measles, severe acute respiratory syndrome, and West Nile virus.

The results of early clinical trials of DNA vaccine candidates indicated favorable safety and tolerability profiles and evidence of humoral and cell-mediated immune responses [7,8]. However, the perceived need by most investigators to improve the immunogenicity of DNA vaccines resulted in the development of strategies to enhance DNA vaccine performance in humans. These strategies fall into four non-mutually exclusive categories, including: (**1**) plasmid design (e.g., codon optimization, promoter selection, inclusion of a genetic-adjuvant-encoding plasmid); (**2**) formulations (e.g., polymer, cationic liposomes, PLGA microspheres); (**3**) devices (e.g., Biojector^®^ 2000, particle-mediated epidermal delivery, electroporation); and (**4**) heterologous prime-boost with a viral vector (e.g., NYVAC, MVA, adenovirus) or recombinant protein. Vical Incorporated (hereafter Vical) developed a CMV DNA vaccine candidate utilizing the first two of these strategies. Proof-of-concept testing of the vaccine in a phase 2 trial has been completed and the vaccine is expected to enter a pivotal phase 3 trial sponsored by Astellas Pharma, Incorporated (hereafter Astellas). A case study is presented here of the development pathway of this vaccine.

### 1.2. CMV Background, Unmet Medical Needs, and Previous Vaccine Development Efforts

Cytomegalovirus, a β-herpesvirus and the largest virus known to infect humans, initiates a predominantly asymptomatic infection in normal, healthy individuals that persists for life, mostly as a latent infection without evidence of viremia [9,10]. CMV infection rates are high worldwide; the CMV seroprevalence rate in the U.S. is ~60% in ≥6 year olds and increases with age [11]. CMV can cause significant morbidity and mortality in certain high-risk situations including congenital infection of fetuses and infection of recipients of hematopoietic stem cell transplant (HCT) or solid organ transplant (SOT), where treatment-related immunosuppression provides opportunities for CMV replication following viral acquisition or reactivation from latency. Antiviral drugs are licensed for use in transplant recipients either prophylactically or preemptively (upon evidence of viremia as measured by PCR or antigenemia assay). Unfortunately, the use of first line ganciclovir-based drugs or second line foscarnet and cidofovir can result in substantial hematotoxicity and nephrotoxicity [9]. While antiviral drugs have reduced the incidence of CMV end organ disease (EOD) in CMV^+^ HCT recipients from 25% prior to their licensure to approximately 5% today [9,12], alternative measures for controlling CMV replication after transplantation without attendant drug toxicities are needed. Vaccines represent one such strategy.

Numerous phase 1 and phase 2 trials have been conducted with several CMV vaccine candidates but a vaccine has yet to be licensed for any indication [10]. Vaccine candidates include live attenuated vaccines such as Towne strain and Towne/Toledo chimeric strains, subunit vaccines, most notably adjuvanted recombinant gB, and vectored vaccines including recombinant viral vectors using canarypox and alphavirus platforms, and plasmid DNA vaccines [10]. The live attenuated CMV Towne vaccine, derived in 1975, provided proof of concept in SOT recipients for reduced CMV disease severity after transplantation; however, it has not provided efficacy against infection in transplant recipients or in women exposed to CMV in daycare settings, and further development of this vaccine appears to depend upon implementing strategies to improve its immunogenicity [10]. Beginning in the 1990s, a recombinant gB vaccine produced in CHO cells was developed and tested in combination with an MF59 adjuvant in multiple clinical studies over the ensuing decades. Two recent randomized controlled phase 2 studies provided proof of concept for the importance of gB as a protective CMV antigen by demonstrating 50% efficacy in decreasing maternal CMV infection [13] and a significant reduction in the duration of viremia as well as the duration of antiviral ganciclovir treatment in CMV seronegative (CMV^−^) recipients of kidneys or livers from CMV^+^ donors [14]. Despite these results it is unclear whether gB adjuvanted by MF59 will continue further development for either indication. Other vaccine approaches such as viral vectored gB [15] and/or pp65 [16,17] have completed phase 1 testing with evidence of safety and immunogenicity. It is conceivable that the recent recognition of the potential importance of creating vaccines that target the pentameric gH/gL/UL128/UL130/UL131 epithelial entry pathway may have contributed to redirected vaccine efforts by some companies, at least for prophylactic vaccines attempting to block both fibroblast (gB-mediated) and epithelial entry pathways [10].

## 2. CMV DNA Vaccine Product Development

### 2.1. Functional Areas

**Figure 1:**
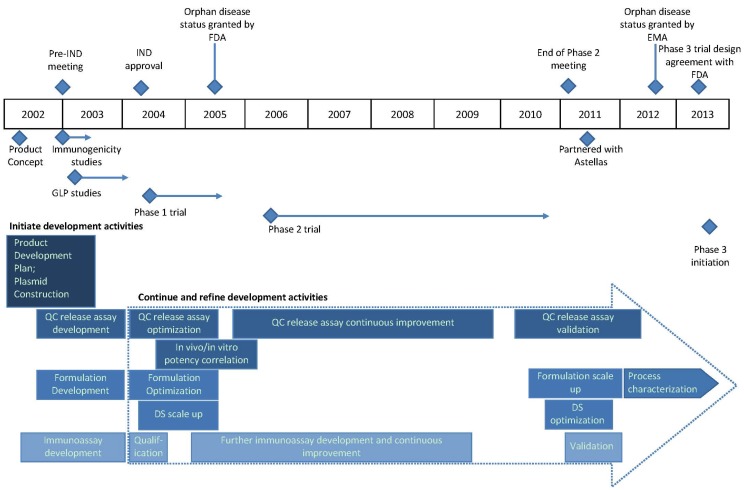
Clinical development timeline of ASP0113 (TransVax) from product concept in 2002 to initiation of a phase 3 trial in 2013. Initiation of various activities is shown in blue diamonds; regulatory activities are shown above the timeline and all others below the timeline. Horizontal blue lines and arrows depict the duration of the indicated activities. Development activities that continued and/or were refined during clinical development are shown in rectangular boxes within the large dotted arrow. Abbreviations: IND, investigational new drug application; FDA, U.S. Food and Drug Administration; EMA, European Medicines Agency; GLP, good laboratory practices; QC, quality control; DS, drug substance.

Vical built the capabilities for developing plasmid DNA-based products during development of its lead product, an intralesional immunotherapeutic called Allovectin^®^ (velimogene aliplasmid) [18], which is currently being evaluated in a phase 3 metastatic melanoma trial that is nearing completion. In the early 2000s, product development activities began on several infectious disease targets, the first being CMV; by that time all of the key functional areas were in place to design, create, manufacture, release, and clinically test DNA vaccine product candidates. The functional area expertise included vaccine research, molecular biology, pharmaceutics, nonclinical testing, manufacturing, assay development, quality control (QC), quality assurance, clinical research and operations, regulatory affairs, and project planning and management. As described below, the initial CMV vaccine target indication was for CMV^+^ HCT recipients. A product development timeline with all supporting activities for this vaccine is displayed in Figure 1 and described in the following sections. The vaccine has been referred to by different names throughout its development history including VCL-CB01, TransVax™, and ASP0113, the current designation of this vaccine since its license to Astellas in 2011.

### 2.2. Vaccine for HCT Recipients as Initial Target Indication

CMV-seropositive recipients of an allogeneic HCT have high attack rates of CMV reactivation following transplantation: 50% to 70% will develop detectable CMV viremia within the first 100 days [19] and are at increased risk for developing CMV EOD if antiviral therapy is not initiated. Due to the overall high CMV-seroprevalence rate, CMV^+^ subjects represent a majority of subjects who undergo HCT. The original intent was to vaccinate normal healthy HCT donors with a 3 dose vaccination series to prime or boost CMV immune responses and then to vaccinate recipients at the time of transplantation to boost the donor’s cells. This dosing regimen was thought to be superior to dosing only the recipient at the time of transplantation because of the potential reduction in vaccine efficacy due to the immunosuppressive medications required post-transplant. After initial testing in CMV^+^ HCT recipients, Vical planned subsequent evaluation of the same vaccine product in CMV^−^ SOT recipients of organs from CMV^+^ donors (D^+^/R^−^), who represent approximately 20% of all solid organ transplantations and those at highest risk for CMV infection and disease.

There are several key advantages that plasmid DNA vaccines can offer relative to other vaccine approaches for application in transplant recipients. First, there is a considerable safety advantage compared to live attenuated virus or viral vectors which may replicate to undesirable levels in an immunosuppressed setting typical of myeloablative or nonmyeloablative conditioning prior to transplantation. Second, there is the ability to stimulate the desired antiviral T-cell responses like live virus vaccines but unlike protein vaccines. Third, repeated vaccine administration would be required for a vaccine in this population, which is readily accomplished with plasmid DNA vaccines, but which may be difficult with viral vector vaccines which typically invoke antivector immune responses during repetitive injections. Fourth, DNA vaccines permit a focusing of immune responses on select antigens in addition to avoidance of immunoevasive CMV proteins coincidentally expressed by live attenuated CMV strains. A DNA vaccine therefore offers the potential for T-cell inducing qualities reminiscent of a live virus vaccine with the safety profile of a protein subunit vaccine—an ideal vaccine profile for transplant recipients.

### 2.3. Antigen Selection

Vical initiated development of a CMV DNA vaccine for transplant recipients in 2002 by first selecting putatively protective CMV antigens and constructing the corresponding plasmids. The control of CMV replication in transplant recipients was predominantly attributed to both CD8^+^ and CD4^+^ T-cell responses but a role for a vaccine-induced antibody response in reducing CMV viral loads could not be excluded and could be important in high risk CMV^−^ SOT recipients who receive an organ from a CMV^+^ donor. Based on the prevailing knowledge at that time, dominant T- and B-cell antigens recognized after natural infection were identified as vaccine candidates including a tegument phosphoprotein, pp65, a dominant T-cell antigen and an envelope glycoprotein, gB, a major target for neutralizing antibodies [20,21,22]. A comprehensive study was subsequently published in 2005 in which the prevalence of both CD4^+^ and CD8^+^ T-cell recognition of essentially all (213) CMV proteins was characterized in 33 CMV^+^ normal, healthy subjects; the results further supported the prevalent recognition of these antigens [23]. Finally, adoptive transfer studies of CMV-specific T cells supported the hypothesis that the antiviral activity of pp65-specific T-cells can control CMV replication in transplant recipients [24,25,26,27]. The DNA vaccine concept was to elicit both humoral and T-cell mediated immune responses to key CMV antigens that could provide immune control early after allogeneic transplantation when CMV^+^ recipients face the highest risk of CMV reactivation and EOD, namely within the first 100 days.

### 2.4. Plasmid and Genetic Construct Designs

The sequences of the CMV antigens encoded by the plasmids were derived from the laboratory-adapted CMV strain AD169 [28]. Prior to gene synthesis and cloning, the protein sequences for both gB and pp65 were modified *in silico*. A secreted form of gB was created (713 amino acids in lieu of the full-length 906 amino acids) that could retain conformationally-intact epitopes and provide higher antibody titers than a nonsecreted form [29]. Four amino acids, ^435^RKRK^438^, were deleted from the otherwise full-length, 561 amino acid, pp65, thereby eliminating a putative kinase site [30] and mitigating theoretical safety concerns of expressing wild-type pp65. Once the final protein sequences were established, a codon-optimization using an algorithm developed at Vical was performed by changing the codon usage of five amino acids to more frequently used codons (U.S. patent 7,410,795). Each codon-optimized gene was synthesized and cloned into the Vical plasmid backbone VR10051, containing a human CMV IE1 promoter/enhancer and intron A and a modified rabbit beta globin polyA/terminator [28]. The final plasmid nomenclatures were VCL-6365 and VCL-6368 for gB- and pp65-expressing plasmids, respectively.

### 2.5. Formulation

A synthetic, nonionic triblock poloxamer, CRL1005, was selected as the lead formulation candidate for this product. This poloxamer consists of a polyoxypropylene (POP) core flanked on each side by a polyoxyethylene (POE) block. CRL1005, developed by CytRx Research Laboratories (Atlanta, GA), had been evaluated in clinical studies with protein-based vaccines and was found to have a favorable safety profile at doses up to 75 mg/injection [31]. Of direct relevance to DNA vaccines, more recent studies revealed that injection of rhesus macaques with an HIV-1 gag-encoding plasmid formulated with CRL1005 provided the highest T-cell responses compared to other formulations following boosting with an adenovirus-5 expressing gag [32]. Subsequently these investigators incorporated a cationic surfactant, benzalkonium chloride (BAK) capable of binding both CRL1005 and DNA resulting in nanoparticle formation; they demonstrated increased T-cell responses to an HIV-1 gag plasmid with this formulation compared to DNA with CRL1005 or DNA alone [33,34]. BAK, at the concentration used with CRL1005, is an inactive ingredient used in a variety of injectable (e.g., intramuscular, intradermal and other routes) FDA-approved products and as such is not considered a new raw material [35].

The combination of CRL1005 and BAK self-assembles into stable nanoparticles. The formulation appearance is a milky white suspension at room temperature that becomes clear as the temperature drops below the CRL1005 cloud point (5 °C–7 °C). The final bivalent vaccine formulation was designed to be a 1-mL intramuscular (IM) injection consisting of a 5-mg total plasmid dose (2.5 mg of each plasmid), 7.5 mg of CRL1005, and 0.1 mg/mL of BAK, all dissolved in phosphate-buffered saline (PBS) and stored frozen [36]. This formulation provided key practical advantages compared to an alternative cationic lipid-based formulation undergoing development during that time: A higher DNA dose could be formulated (up to 5 mg/mL) and a single vial formulation was possible, in contrast to a multivial format (up to 1 mg/mL) with reconstitution of dried lipid film and mixing for the cationic lipid formulation.

## 3. Nonclinical Studies

### 3.1. *In Vivo* Immunogenicity and Expression Studies

Because of the species tropism of human CMV, no animal challenge model of efficacy exists for testing human CMV vaccine candidates. Instead, immunogenicity testing was conducted in BALB/c mice to characterize the gB-binding serum IgG levels and the frequency of pp65-specific IFN-γ producing-T cells as measured by ELISPOT assay with overlapping peptides [28]. Two-dose and three-dose regimens of the bivalent plasmids and each monovalent plasmid were tested with and without CRL1005/BAK formulation. This formulation was found to significantly increase the immune responses compared to the bivalent vaccine in PBS only, thereby abrogating the immunological interference that was encountered when the combination was prepared in PBS only [28].

Additional *in vivo* studies were conducted in conjunction with a more detailed physical characterization of this formulation [36]. These studies utilized a plasmid encoding a model antigen, influenza A nucleoprotein (NP), which elicited antibody responses and CD4^+^ and CD8^+^ T-cell responses to defined epitopes. Following a three-dose immunization schedule, DNA/CRL1005/BAK-formulations produced significantly higher responses than the same dose of DNA alone; NP antibody responses were 1.6-fold higher (*p* < 0.001), CD4^+^ responses to defined class II-restricted peptides were 1.7-fold higher (*p* < 0.01) and CD8^+^ T-cell responses to a class I-restricted peptide was 1.9-fold higher (*p* < 0.01; [36]). *In vivo* expression studies were also conducted to determine the degree by which this formulation enhanced delivery. Mice received a single injection of plasmid encoding reporter transgenes luciferase (cytoplasmic protein) or erythropoietin (secreted protein). DNA/CRL1005/BAK-formulations compared to DNA alone provided a 3-fold increase in luciferase in muscles and a 5-fold increase in erythropoietin in muscles as well as serum [36]. Collectively these results support the hypothesis that increased immunogenicity can be attributed at least in part to enhanced expression of plasmid with this delivery system.

### 3.2. Safety-Toxicology Study

The first of two good laboratory practice (GLP)-compliant nonclinical studies conducted with the vaccine ASP0113 was a repeat dose safety-toxicology study in rabbits that received 4 IM injections at 2-week intervals of 0 mg, 0.5 mg, and 5 mg doses of product. This study was designed to evaluate clinical signs including injection site reactions (Draize scores), body weights, food consumption, ophthalmological exams, and mortality, as well as clinical pathology including hematology, coagulation, and clinical chemistry panels and anti-nuclear antibodies. No product-related changes in these evaluations were found with the exception of increased but reversible creatinine phosphokinase levels after the last injection and minimal to moderate inflammation in muscle and skin encompassing the injection site, which largely resolved during the recovery period. These are expected observations for IM injected vaccines.

### 3.3. Biodistribution/Integration Study

The second of two GLP-compliant nonclinical studies conducted with ASP0113 was a single dose biodistribution/integration study in mice. Various tissue samples were collected at days 2, 14, 28, and 61 after injection of 100 μg of bivalent product to assess the tissue distribution and clearance kinetics of plasmid over time and compared these results with a control plasmid (formulated in PBS only) previously tested in GLP and clinical studies. Plasmid copies cleared rapidly after injection such that by day 28 the highest copies were found in injection site muscle and with detectable levels only in spleen and bone marrow. There were no significant differences in the plasmid-copy clearance between the two test articles over 61 days. An integration study was conducted and the results supported the conclusion that the risk of plasmid integration was negligible. Pharmacodynamics and pharmacokinetic studies were not required.

## 4. Manufacturing, Formulation/Fill/Finish, and Product Release

### 4.1. Manufacturing of Bulk Drug Substances

Prior to the production of ASP0113, Vical had acquired extensive chemistry, manufacturing, and control (CMC) experience in developing plasmid DNA-based products for clinical testing. For clinical testing of ASP0113, Vical produced all bulk drug substance (DS) lots of each of the CMV plasmids according to current good manufacturing practices (cGMP). Each plasmid was produced by bacterial fermentation using *E. coli* strain DH10B under kanamycin selection. A master cell bank was created for each plasmid with specifications for purity, potency, and identity. A manufacturer’s working cell bank was derived from the master cell bank and used to inoculate an overnight culture which in turn was used to inoculate a fermentor (initially 100 L and later 500 L scale). Bacterial cell paste was collected following fermentation and was processed by alkaline lysis followed by filtration and precipitation procedures. Following downstream chromatography steps, the final purified bulk DS for each plasmid was adjusted to the final DNA concentration and frozen. In-process QC testing was conducted throughout all of the above steps. Aliquots from the final bulk DS lots were placed on a 36-month stability program and other samples were submitted for release testing that incorporates measures of purity, strength/potency, and identity. Vical’s DS release specifications, including tests for the host *E. coli* macromolecules, genomic DNA, RNA, and protein (all <1%), endotoxin <40 EU/mg DNA, and percent of supercoiled plasmid DNA at >80% follow the acceptance criteria recommended in the 2007 FDA Guidance for Industry document for plasmid DNA vaccines.

### 4.2. Formulation/Fill/Finish and Release of Drug Product

The final drug product (DP) was manufactured by mixing each monovalent bulk DS at a 1:1 mass ratio to create a bivalent bulk DS that was then formulated with CRL1005 and BAK under aseptic conditions, resulting in the bulk DP. A challenging aspect of the formulation development was the requirement to maintain the temperature of the CRL1005-containing solution below the cloud point during filtration. During early development the bulk DP was sterile filtered below the cloud point. However, significant product losses were incurred, leading to optimization of the process prior to phase 3. Sterile filtration of a premix containing BAK and CRL1005 below the cloud point was implemented, followed by introduction of sterile-filtered bulk DS to form the bulk DP.

Bulk DP was aseptically filled into glass vials and stored frozen. Samples of DP were tested for release as well as placed on a 36-month stability program; both DS and DP achieved at least 36 months of stability. The current DP release tests used for purity include appearance, endotoxin, sterility, percent supercoiled plasmid DNA, particle size, general safety test, and particulate matter. The release tests used for identity include pH, total DNA size, and Western blots for the products of each plasmid. The majority of the DS and the DP release assays are standard, *i.e.*, applicable regardless of plasmid. However, one plasmid-specific DP release assay, a relative potency assay, merits discussion for its importance as a robust assay for release and stability testing.

A TaqMan^®^-based reverse-transcriptase, polymerase chain reaction (RT-PCR) assay was developed which measures the mRNA expression of both gB and pp65 following transfection of the bivalent product, as an indication of the product’s *in vitro* potency [37]. Messenger RNA expression from plasmid DNA is the most immediate biological activity measurable after DNA vaccine delivery. This highly-specific assay was designed to detect expression of each of the plasmid-derived transgenes in comparison to a reference standard to establish the percent relative potency (%RP) of any test sample. The %RP assay was incorporated as a DP release test for phase 2 clinical trial material (CTM) lots as well as for measuring stability of each lot and lot-to-lot consistency. Furthermore, a correlation was established between *in vitro* potency and *in vivo* gB antibody responses in mice with samples subjected to forced degradation by heat; the %RP assay proved to be an excellent indicator of *in vivo* potency [36].

By late 2003, Vical had completed all of the appropriate activities and compiled the requisite documentation for filing an investigational new drug application (IND) with the U.S. FDA. IND allowance occurred in early 2004 followed shortly by initiation of a phase 1 trial.

## 5. Clinical Trials

### 5.1. Phase 1

#### 5.1.1. Trial Design and Safety in Normal Healthy Subjects

The first-in-human clinical testing of ASP0113 (denoted then as VCL-CB01) was a multicenter open label phase 1 trial in 44 normal, healthy adults (22 each CMV^+^ and CMV^−^) age 18–45 years to evaluate the safety and immunogenicity of the bivalent vaccine [38]. Subjects received IM injections of 1-mg or 5-mg DNA doses on a 0-, 2-, and 8-week schedule or 5-mg DNA dose on an accelerated 0-, 3-, 7-, and 28-day schedule.

In that setting, the vaccine was well tolerated, with no serious adverse events (SAEs) and no discontinuations due to vaccine-related adverse events (AEs). The most frequent AEs were injection site pain, myalgia, headache, and malaise and were of mild to moderate severity. Systemic reactions included mild to moderate malaise and myalgia. Local reactions included injection site pain, induration, swelling, and erythema [38].

#### 5.1.2. Immunogenicity Findings

T-cell responses through Week 16 were assessed in an *ex vivo* ELISPOT assay using pp65 or gB overlapping peptides [38]. Antibody responses to gB were assessed in an indirect binding ELISA using full-length recombinant gB protein isolated from transfected CHO cells. T-cell and/or antibody responses to vaccine encoded antigens were elicited in 37.5% and 50% of the CMV^-^ subjects in the 1-mg and 5-mg dose groups, respectively, suggesting a possible dose response; however, the number of subjects in each group was not powered to detect a difference in the response rates for the different doses and vaccination schedules. T-cell responses to pp65 were detected in 12.5% to 37.5% of CMV^+^ subjects, but no CMV^+^ subject in any group had greater than a 2-fold increase in gB antibody levels, suggesting that the antibody responses were not boosted by the vaccine.

To evaluate the duration of the T-cell responses in CMV^-^ subjects, the *ex vivo* ELISPOT assay was performed with Week 32 specimens from CMV^−^ subjects on the 0-, 2-, 8-week injection schedule. Neither of the 2 subjects in the 1-mg dose group who were responders in the assay at earlier time points had detectable responses at Week 32. Conversely, in the 5-mg dose group, 5 subjects (62.5%) had detectable responses, including all 3 of the subjects who had T-cell responses by Week 16, and an additional 2 subjects who did not have detectable responses by Week 16 (*p* = 0.0256; Fisher’s exact test, for responses in the 1-mg and 5-mg dose groups at Week 32). These results suggested a dose response for CMV^−^ subjects, even though a dose response was not established in the initial evaluation of the T-cell responses through Week 16.

T-cell responses at Week 32 in CMV^−^ subjects on the 0-, 2-, 8-week injection schedule and at Week 16 for those on the 0-, 3-, 7-, 28-day schedule were also evaluated by the cultured ELISPOT assay, which demonstrates the ability of vaccine-primed memory T cells to proliferate and produce IFN-γ on exposure to vaccine encoded antigen. In the assay, peripheral blood mononuclear cells (PBMC) were cultured with pp65 and gB peptides and recombinant human IL-2 for 10 days prior to evaluation in the IFN-γ ELISPOT assay. Altogether, vaccine-primed memory T-cells were detected in 15 of 22 CMV^−^ subjects (68%) [38]. Moreover, memory T-cell responses were detected in 5 CMV^−^ subjects who failed to demonstrate T-cell responses in the *ex vivo* ELISPOT assay at any time point indicating that priming of memory T-cell responses had occurred even in the absence of a detectable effector T-cell response.

### 5.2. Phase 2

#### 5.2.1. Trial Design and Safety in HCT Recipients

The second clinical trial of ASP0113 was a multicenter, randomized, double-blind, placebo-controlled phase 2 trial in CMV^+^, allogeneic HCT recipients aged 18–65 years with various forms of lymphoma and leukemia [39]. According to the original trial protocol 80 donor-recipient pairs undergoing HCT were planned for enrollment but donor vaccination proved logistically impractical and the protocol was amended to enroll a recipient-only (unpaired) arm. Subjects were randomized 1:1 to receive 5-mg doses of ASP0113 or PBS placebo prior to ablative conditioning and at approximately 1, 3, and 6 months after transplantation and were stratified by clinical site, donor-recipient human leukocyte antigen match, and by donor CMV serostatus. All subjects who received at least one dose were included in the safety analysis and a total of 74 subjects were included in the per protocol (PP) population analysis. The two groups were well-balanced for demographics, conditioning regimens, and donor relatedness and serostatus.

All HCT recipients developed at least 1 treatment-emergent adverse event and the majority (>70%) of subjects in both groups developed an SAE during the study, which was not statistically different between groups. The incidence of local reactogenicity was higher in the ASP0113 group than in the placebo group (22.9% and 10.9%, respectively), primarily due to injection site pain. One SAE in the vaccine group, an allergic reaction which resolved after treatment, was deemed possibly related by the Sponsor. Overall, the safety profiles of both groups were similar and the vaccine was considered well tolerated in this trial [39].

#### 5.2.2. Efficacy

The primary efficacy endpoint, the rate of initiation of CMV-specific antiviral therapy was lower in the PP population for the vaccine group (19/40, 47.5%) compared to the placebo group (21/34, 61.8%) but the difference did not achieve statistical significance (*p* = 0.145). Considerable variability existed among the 16 trial sites in the types of local laboratory assays used for detecting CMV and each institute had different treatment algorithms for initiating preemptive antiviral therapy. In contrast, measurement of plasma levels of CMV DNA (also referred to as CMV viremia) using a quantitative PCR assay performed at a single, central laboratory revealed statistically significant differences between the two groups. Compared to the placebo group, the vaccine group had significantly lower occurrence of detectable CMV viremia (*p* = 0.008), fewer CMV viremic episodes (*p* = 0.017), longer time to initial viremia (*p* = 0.003), and shorter duration of viremia when normalized to days on study (*p* = 0.042).

Several secondary endpoints representative of the clinical manifestations attributed directly or indirectly to CMV infection were numerically lower in the vaccine group compared to the placebo group but did not achieve significance; however, this study was not powered to detect differences in these endpoints. These endpoints included CMV EOD (manifest as pneumonia or gastroenteritis), overall mortality, grade 3–4 acute GVHD, and severe chronic GVHD; a post-hoc analysis of a composite of these endpoints revealed a difference in favor of vaccine which did not achieve statistical significance (*p* = 0.10). Based on the phase 2 findings and as described further in Section 5.3, overall mortality was selected for inclusion into the primary endpoint for a pivotal phase 3 trial, with the prospect for also including several other endpoints as a composite.

#### 5.2.3. Immunogenicity Findings

T-cell responses to pp65 and gB for HCT recipients in the ASP0113 and placebo groups were assessed in the direct *ex vivo* IFN-γ ELISPOT assay and levels of gB-specific antibody were assessed in the indirect binding ELISA [39]. T-cell responses and antibody levels were similar in the ASP0113 and placebo recipients prior to transplant. However, donor-derived T-cell responses to pp65 or gB were not evaluated, so an impact of imbalances in these T-cell responses cannot be ruled out.

T-cell responses to pp65 were numerically higher in the ASP0113 group relative to the placebo group at all of the time points evaluated after transplant. Despite the variability in the magnitude of the responses, there was a trend towards a significant difference in the responses at Day 56, and statistical significance was reached by Day 84 (*p* = 0.075 and 0.036, respectively; Wilcoxon rank-sum test). In a post-hoc analysis of the T-cell responses to pp65, an ordinal logistic regression model was used because the T-cell responses had a U-shaped distribution in both groups [40]. Three categories, defined as <750, 750–2,999, and ≥3,000 SFU/10^6^ PBMC (with boundaries at ½ and 2 times the mean of approximately 1,500 SFU/10^6^ PBMC in normal CMV^+^ individuals), were used for analysis of the pp65 responses. The treatment effect p-value of 0.022 for Day 56 through Day 365, suggests that recipients of ASP0113 were significantly more likely to have high levels of pp65 T cells compared with the placebo group. This finding was reflected in the mean values of pp65-specific T cells which were more than twice the mean for normal CMV^+^ adults (1500 SFU/10^6^ PBMC). The mean T-cell responses to gB were also numerically higher in the ASP0113 group than in the placebo group at all time points after Day 84; however, the differences at any time point were not significant.

The pp65 T-cell responses in the ASP0113 group could have been enhanced by ablative conditioning prior to transplant, which may have created “immunological space” for the rapid expansion of CMV antigen-specific T-cells upon exposure to vaccine encoded antigens. Because expansion could also occur in either group as a result of exposure to CMV during viral reactivation, in a second post-hoc analysis using the ordinal logistic regression model, the pp65 T-cell responses were censored after the occurrence of viremia to eliminate the influence of exposure to CMV. The pp65 T-cell responses in the ASP0113 group were still higher than those of the placebo group with a treatment effect of *p* = 0.005, suggesting that the comparatively higher responses in the ASP0113 group were independent of CMV viremia [40] and likely due to vaccination with ASP0113. Furthermore, for those subjects who did not have CMV viremia by Day 56, the pp65 T-cell responses at Day 56 were significantly higher in the ASP0113 group than in the placebo group (*p* = 0.030; Wilcoxon rank-sum test), indicating that the divergence of the responses in the two groups begins early in the vaccination regimen, providing protection from CMV reactivation during the time period when most CMV viremia occurs.

The geometric mean gB antibody levels were not significantly higher in the ASP0113 group than in the placebo group until after the fourth injection, when the gB antibody levels showed a trend towards significance at Day 210 and reached significance by Day 365 (*p* = 0.064 and 0.009, respectively; Wilcoxon rank-sum test). The late divergence of the gB antibody levels in the two groups compared with the early divergence of the T-cell responses may reflect differences in both the persistence of antibodies and T cells after ablation and the kinetics of their recoveries after transplantation.

### 5.3. Phase 3 Trial in HCT Recipients

The design of a global phase 3 trial of ASP0113, sponsored by Astellas Pharma Global Development, Inc. with Vical as collaborators, has been recently registered at www.clinicaltrials.gov. The official title of this protocol, 0113-CL-1004, is “A Randomized, Double-Blind, Placebo-Controlled, Phase 3 Trial to Evaluate the Protective Efficacy and Safety of a Therapeutic Vaccine, ASP0113, in Cytomegalovirus (CMV)-Seropositive Recipients Undergoing Allogeneic Hematopoietic Cell Transplant (HCT)”. Subjects will be randomized 1:1 to receive ASP0113 or PBS placebo. The primary efficacy endpoint of this trial is overall mortality at one year post-transplantation. The safety of ASP0113 in HCT recipients will also be monitored. This trial is anticipated to enroll 500 subjects and is analytically divided into two parts, with Part 1 enrolling 100 subjects and Part 2 enrolling 400 subjects. Part 1 will be used to evaluate the adequacy of the primary endpoint and, if necessary, to modify the primary endpoint to be specified for Part 2. This trial will enroll subjects at approximately 90 HCT centers in North America, Europe, Asia, and Australia and is anticipated to be completed in September of 2016.

## 6. Process Improvements, Manufacturing Scale-Up, and Validation of Assays

During production of DP for testing in phase 1 and phase 2 trials, improvements in CMC-related processes and refinements in analytical testing methods and assay specifications were identified and implemented prior to phase 3 testing. Furthermore, the DP lot sizes sufficient for phase 1 and phase 2 CTMs had to be increased by scaling up the manufacturing procedures to meet the CTM demands for phase 3. Process improvements enhanced manufacturing efficiencies and lot-to-lot consistency by streamlining/simplifying procedures, upgrading equipment, and enriching product yield. Some assays underwent refinements that resulted in increased sensitivity, precision, ease of use, and reagent stability and availability. Each plasmid-specific DS and DP release assay was validated in preparation for use in phase 3. All of the CMC-related changes were submitted to the FDA under the current IND in 2012.

## 7. Timeline Summary of ASP0113 Development History

The timeline in Figure 1 integrates all of the key activities described above and illustrates both the typical and unanticipated factors that can impact product development timelines. A reasonably representative time frame ensued from product concept to completion of phase 1 testing and accordingly the indication of the favorable safety profile and demonstration of immunogenicity in humans [38,41], a vital first stage for a vaccine program. In contrast, the phase 2 trial in HCT recipients took an extended period of time to enroll, largely due to the novelty of the approach and the inherently-complex nature of conducting a vaccine trial in subjects with such underlying conditions and treatment-associated morbidity; furthermore, as described in Section 2.2, the original premise was to vaccinate donors but due to the limited time donors were available prior to transplant, this proved impractical and this arm was discontinued while leaving the recipient-only arm open. In a strategy to mitigate some of the risks inherent in product development, most optimization and assay validation activities were delayed until late into the phase 2 trial. Ultimate agreement with the FDA, in addition to discussions with other global regulatory agencies in Europe and Asia, required thorough and frequent communications to define acceptable endpoints for licensure. Vical licensed this program to Astellas during this period, with joint participation from both groups for regulatory discussions on phase 3 trial endpoints. While some aspects of this product development history could have been shortened, small biotechnology companies such as Vical typically encounter resource constraints and risk management plays an important role in the timing of activities. Fortunately, the partnership for continued development of ASP0113 provides a tremendous opportunity for advancing this first-in-class therapeutic CMV vaccine towards product licensure.

## 8. Conclusions—Additional CMV DNA Vaccine Opportunities

The proof of concept findings for the bivalent CMV vaccine in HCT recipients provides support for testing this product in SOT recipients. A randomized, double-blind, placebo-controlled phase 2 trial is planned with ASP0113 in CMV^−^ recipients receiving a kidney transplant from either a living donor or a deceased donor who is CMV^+^, the highest risk group among SOT recipients for experiencing CMV disease.

Finally, a CMV prophylactic vaccine that can prevent congenital CMV infection would have a tremendous public health impact. However, the development of a vaccine for this indication will require considerable resources due to a longer clinical development pathway and potentially high numbers of subjects for clinical trials. Furthermore, the clinical endpoint required for licensure of such a vaccine has not been finalized [42] and has only recently received discussion between government, academia, and manufacturers [43]. Vical initiated development of a prophylactic congenital CMV vaccine (CyMVectin™) by developing a different product formulation, a cationic lipid adjuvant called Vaxfectin^®^, combined with gB and pp65-expressing plasmids and recently published nonclinical immunogenicity and GLP safety studies [44]. Given the enormous scope of developing such a vaccine, further development of CyMVectin™ would ideally proceed in partnership with a large pharma company. In parallel, additional research is ongoing to identify one or more additional plasmids expressing other CMV genes (Section 1.2) for potential inclusion in this vaccine. The encouraging proof of concept results of ASP0113 in the difficult HCT recipient population provides optimism for developing CMV vaccines for additional unmet needs. 
